# Decentral gene expression analysis for ER+/Her2− breast cancer: results of a proficiency testing program for the EndoPredict assay

**DOI:** 10.1007/s00428-012-1204-4

**Published:** 2012-02-28

**Authors:** Carsten Denkert, Ralf Kronenwett, Werner Schlake, Kerstin Bohmann, Roland Penzel, Karsten E. Weber, Heinz Höfler, Ulrich Lehmann, Peter Schirmacher, Katja Specht, Margaretha Rudas, Hans-Heinrich Kreipe, Peter Schraml, Gudrun Schlake, Zsuzsanna Bago-Horvath, Frank Tiecke, Zsuzsanna Varga, Holger Moch, Marcus Schmidt, Judith Prinzler, Dontscho Kerjaschki, Bruno Valentin Sinn, Berit Maria Müller, Martin Filipits, Christoph Petry, Manfred Dietel

**Affiliations:** 1 Institute of Pathology, Charité Hospital, Campus Mitte, Schumannstr. 20/21, 10117 Berlin, Germany; 2 Sividon Diagnostics GmbH, 50829 Cologne, Germany; 3 Institute of Pathology, 45879 Gelsenkirchen, Germany; 4 Institute of Pathology, University of Heidelberg, 69120 Heidelberg, Germany; 5 Institute of Pathology and Pathological Anatomy, Technical University of Munich, 81675 Munich, Germany; 6 Institute of Pathology, Medizinische Hochschule Hannover, 30625 Hannover, Germany; 7 Institute of Pathology, Medical University of Vienna, 1090 Vienna, Austria; 8 Institute of Surgical Pathology, University Hospital Zurich, 8091 Zurich, Switzerland; 9 Department of Gynecology and Obstetrics, University of Mainz, Mainz, Germany; 10 Institute of Cancer Research, Medical University of Vienna, 1090 Vienna, Austria

**Keywords:** Breast cancer, Prognosis, mRNA, Quality control

## Abstract

Gene expression profiles provide important information about the biology of breast tumors and can be used to develop prognostic tests. However, the implementation of quantitative RNA-based testing in routine molecular pathology has not been accomplished, so far. The EndoPredict assay has recently been described as a quantitative RT-PCR-based multigene expression test to identify a subgroup of hormone–receptor-positive tumors that have an excellent prognosis with endocrine therapy only. To transfer this test from bench to bedside, it is essential to evaluate the test–performance in a multicenter setting in different molecular pathology laboratories. In this study, we have evaluated the EndoPredict (EP) assay in seven different molecular pathology laboratories in Germany, Austria, and Switzerland. A set of ten formalin-fixed paraffin-embedded tumors was tested in the different labs, and the variance and accuracy of the EndoPredict assays were determined using predefined reference values. Extraction of a sufficient amount of RNA and generation of a valid EP score was possible for all 70 study samples (100%). The EP scores measured by the individual participants showed an excellent correlation with the reference values, respectively, as reflected by Pearson correlation coefficients ranging from 0.987 to 0.999. The Pearson correlation coefficient of all values compared to the reference value was 0.994. All laboratories determined EP scores for all samples differing not more than 1.0 score units from the pre-defined references. All samples were assigned to the correct EP risk group, resulting in a sensitivity and specificity of 100%, a concordance of 100%, and a kappa of 1.0. Taken together, the EndoPredict test could be successfully implemented in all seven participating laboratories and is feasible for reliable decentralized assessment of gene expression in luminal breast cancer.

## Introduction

The success of individualized cancer therapy critically depends on reliable molecular biomarker assays that identify those tumors that have a particular good response to a defined treatment.

In the last years, molecular assays for prediction of therapy response have been established in colon cancer and non-small cell lung cancer. These assays are based on retrospective evaluation of clinical trials that had been performed to evaluate new therapeutic approaches [1, 2]. It has been shown that determination of *EGFR* and *KRAS* mutations in formalin-fixed paraffin-embedded (FFPE) tissues can be performed reliably in the routine molecular pathology laboratory [3, 4].

While this approach is now routine for colorectal and lung cancer, the molecular characterization of breast cancer in the pathology institutes is largely based on immunohistochemical evaluation of hormone receptors and HER2 [5, 6]. However, one central clinical question in breast cancer is the identification of those tumors that have an excellent outcome with endocrine therapy alone—a task which cannot be accomplished by standard immunohistochemistry.

It has been shown in several studies that gene expression analysis can identify subgroups of breast tumors with good outcome under endocrine therapy [7–10]. Based on these observations, molecular assays have been developed that are currently performed centralized in reference laboratories in Europe [11] and the USA [12, 13]. These assays provide useful information for treatment strategies; however, they are not linked to the histopathology workflow in the local pathology laboratory. As most of the tissue-derived information is generated in clinical pathology laboratories, it would improve the acceptance of the new technologies if the molecular assay would be available in each pathology institute that diagnoses the breast cancer cases anyway.

We have recently described a quantitative reverse transcription polymerase chain reaction (RT-qPCR)-based molecular assay that uses routine FFPE tissue samples and identifies a subgroup of breast cancer cases that have an excellent prognosis if treated with endocrine therapy alone, without additional chemotherapy [14]. The assay measures the expression of eight functional genes and three normalization genes as well as the presence of genomic DNA to calculate the EndoPredict score (EP score) ranging from 0 to 15. Using the validated cutoff value of 5, patients can be classified into low or high risk for the occurrence of distant recurrence under endocrine therapy. The molecular score can subsequently be combined with the nodal status and the tumor size to calculate the integrated molecular and clinical risk score (EPclin). The EPclin score is superior over the EP score as the outcome of breast cancer cannot be predicted optimally by gene expression data alone [14]. Clinical parameters reflecting the size and the dissemination status of the tumor are not necessarily reflected by tumor RNA expression.

The EndoPredict score had been generated in a cohort of 964 ER-positive, HER2-negative tumors. After transfer to the RT-qPCR platform, the test was validated independently in two separate clinical cohorts, the ABCSG-6 (*n* = 378), and the ABCSG-8 (*n* = 1,324) cohort [14]. This validation approach resulted in a level of evidence of 1 according to the classification scheme for biomarker studies that has been suggested by Simon et al. [15].

The next and essential step would be to transfer this molecular testing system to the individual clinical pathology laboratories. In this study, we report the results of the proficiency testing program, which show that the EndoPredict test can be executed reliably and de-centralized in molecular–pathological laboratories. Aim of the study was to evaluate the performance of the test in the different molecular pathology laboratories and to determine the number of laboratories that have implemented the EndoPredict test successfully.

## Methods

### Materials and study logistics

**Fig. 1:**
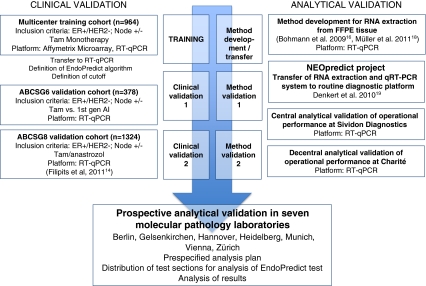
Workflow of sequential validation of the EndoPredict assay and the interlaboratory quality assurance (*ER* estrogen receptor)

Ten-micrometer sections of ten FFPE breast cancer tumor blocks were shipped to the seven participating laboratories: Institute of Pathology, Charité Hospital, Berlin; Institute of Pathology, Gelsenkirchen, Germany; Institute of Pathology, Medizinische Hochschule Hannover, Germany; Institute of Pathology, University of Heidelberg, Germany; Institute of Pathology and Pathological Anatomy, Technical University of Munich, Germany; Institute of Pathology, Medizinische Universität Wien, Vienna, Austria; and Institute of Surgical Pathology, University Hospital Zurich, Switzerland. Selection criteria for these institutions were (1) experience in high-volume diagnostic molecular pathology and breast pathology, (2) localization in different countries (Germany, Austria, or Switzerland); (3) different organizational structure (university hospital or large specialized private institution). The pathological data of the tumors are shown in Table 1, the steps for development of the EndoPredict assay and the workflow of the study are incorporated in Fig. 1.

**Table 1:** Clinicopathological parameters

Sample ID	Tumor content (%)	Grade	pT stage	pN stage	ER (%)	PR (%)
A	60	2	pT2	pN0	100	60
B	75	2	pT2	pN0(sn)	90	10
C	65	3	pT2(m)	pN2a	95	40
D	80	1	pT2	pN0	Pos.	n.d.
E	65	2	pT1c	pN0	100	0
F	75	2	pT2	pN0(sn)	Pos.	n.d.
G	30	1	pT2	pN1mi	100	30
H	70	3	pT2	pN2a	90	80
I	60	2	pT2	pN3a	90	8
J	75	2	pT3(m)	pN1a	95	0

Adjacent sections were used for histopathological quality control to ensure that the sample used for the molecular analysis contains at least 30% of tumor tissue. The range of tumor content of the samples was 30–80%, mean tumor content 63%.

For each block, a reference EP score was generated in Sividon’s laboratory by measurement of four further sections of each tumor. The reference values were calculated as means of these four replicate measurements. The range of the EP scores of the ten tumors was from 2.7 to 12.1. For this analytical evaluation of the EndoPredict assay, the samples were chosen to cover the range of possible EP scores.

These reference EP scores were not provided to the study participants until the study was completed and were only used for the final analysis. A random unique number identified each section; the association between an individual section, and the block of origin was unknown to the study participants.

### Pre-specified analysis plan

Study aims and statistical analysis plan were defined prospectively. The primary aim of the study was to determine the number of pathology laboratories that have implemented the EndoPredict assay successfully. For a successful implementation, it was required that the absolute difference between the local EP score and the reference EP score was below 1.0 EP units for at least nine of ten samples. The predefined secondary aims are shown in Table 2. In the event that the analysis could not be completed due to technical reasons or out-of-specification controls the laboratory could ask for replacement sections. The total number of replacement sections and the number of repeated plates were recorded.

**Table 2:** Predefined study aims and summary of the results

Primary aim		Results
	Determine the number of participating laboratories that successfully implement the EndoPredict test. A successful implementation is achieved if the absolute difference between EP scores determined in the laboratory and the corresponding reference EP scores is below 1.0 EP score units for at least 9 of the 10 samples.	100% (7 of 7)
Secondary aims		
1.	Determine the number and ratio of sections for which the EP score was successfully measured (across all blocks and laboratories).	98.6% (69 of 70)
2.	Determine the (mean) standard deviation of EP scores between laboratories (including one result per block from Sividon). Results per block are summarized by averaging the corresponding variances.	0.25 score units (1.7% of EP score range)
3.	Determine the number and ratio of EP scores deviating more than 2.0 EP score units from the reference EP score across all blocks and laboratories (outliers). The same analysis will be done for deviations of more than 1.0 and 0.5 EP score units.	Deviation of more than 2.0 units:
		0 of 70 samples
		Deviation of more than 1.0 units:
		0 of 70 samples (one sample had a deviation of exact 1.0 score unit)
		Deviation of more than 0.5 units:
		10 of 70 samples (14%)
4.	Calculate the Pearson correlation coefficient between the reference EP scores and the EP scores reported by the participating laboratories (across all blocks and laboratories).	0.994
5.	Calculate the EP classes from the EP scores. Determine the number and ratio of EP classes discordant to the corresponding reference EP classes across all blocks for each laboratory (across all laboratories). Also, report contingency table, kappa statistics, sensitivity, and specificity.	All EP classes were calculated correctly, resulting in a sensitivity and specificity of 100% and a kappa of 1.0.

### RNA isolation from FFPE tissue

Total RNA was extracted from one FFPE tissue section of each of the ten FFPE tumor blocks, respectively, using a silica-coated magnetic bead-based method developed by Siemens Healthcare Diagnostics (Marburg, Germany) as published previously [16–19]. The method was either performed manually or automatically, depending on the robotic equipment available at each site. DNA-free total RNA from one FFPE section was finally eluted with 100 μl of elution buffer. For the assessment of DNA contamination in the respective RNA extract, an HBB-DNA-specific qPCR was included in the EndoPredict assay. Samples were considered to be substantially free of DNA when Ct values above 38 were detected. In case of DNA contamination, samples were manually re-digested by DNase I treatment.

### Gene expression analysis using RT-qPCR

Expression of eight genes-of-interest (*AZGP1*, *BIRC5*, *DHCR7*, *IL6ST*, *MGP*, *RBBP8*, *STC2*, *UBE2C*), three normalization genes (*CALM2*, *OAZ1*, *RPL37A*) as well as the amount of residual genomic DNA (HBB) were assessed by the EndoPredict® assay (Sividon Diagnostics, Cologne, Germany). This assay is configured on a 96-well plate containing primers and FAM/TAMRA-labeled probes dried into the wells. Each EndoPredict plate contains PCR assays for two tumor samples as well as one positive and one negative control for each gene. Genes were measured in triplicates as a necessary means to control for PCR imprecision and to enable outlier removal. Sequences of primers and probes were published previously [14]. Gene expression was assessed by one-step RT-qPCR using the SuperScript III PLATINUM One-Step Quantitative RT-PCR System with ROX (Invitrogen, Karlsruhe, Germany) according to manufacturer’s instructions in a VERSANT® kPCR Molecular System (Siemens Healthcare Diagnostics) with 30 min at 50°C, 2 min at 95°C followed by 40 cycles of 15 s at 95°C and 30 s at 60°C. Mastermix containing 1 μl sample RNA for each well was added to the respective wells. As positive controls of RT-qPCR assays a standardized reference RNA (Stratagene qPCR Human Reference Total RNA, Agilent Technologies, Waldbronn, Germany) was tested for each gene on each plate. As positive control for HBB–DNA PCR assay Human Genomic DNA (Roche Applied Bioscience, Mannheim, Germany) was used. For exclusion of contamination no-template-controls were assessed in parallel as well. Detection of outliers which were defined by a difference of more than three “noise model”-predicted standard deviations from the other replicates, relative expression levels of each gene of interest as well as EP scores were calculated as described previously [14] using a web-based implementation to process analytical PCR results into EP scores which can be found at http://forschung.medizin.uni-mainz.de/epreport/. The amount of input RNA was assessed by the EndoPredict assay using the mean of the Ct values of the three normalization genes as surrogate. RNA input was considered as sufficient if the mean of the ct values of *CALM2*, *OAZ1* and *RPL37A* of a tumor sample was below 28. Before the start of the proficiency testing program, each participant performed a pre-defined analytical validation of the performance characteristics of the EndoPredict assay.

### Evaluation of study results

After completion of the PCR, for each sample, analytical data (raw data of PCR run), calculated EP score as well as classification into low or high risk of distant metastasis were reported to Sividon and to Charité, who performed the data analysis according to the pre-defined analysis plan. After completion of the study, summary results and reference EP scores were disclosed to all participants.

## Results

### Successful implementation of the EndoPredict assay—primary study aim

The EndoPredict test could be successfully implemented in all seven participating laboratories. As defined in the study plan, a successful implementation was achieved, if the absolute difference between EP scores determined in the participating laboratory and the corresponding reference EP scores was below 1.0 EP score units for at least nine of the ten samples. Six of the seven laboratories have reached a difference below 1.0 score units for all ten samples. One laboratory had differences below 1.0 score units for nine of ten samples and a difference of exactly 1.0 score unit for a single sample.

**Fig. 2:**
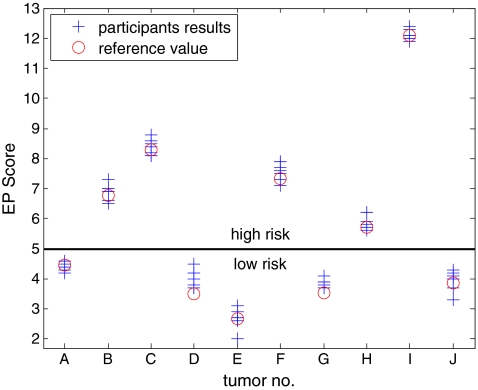
Results of the decentral measurement of 70 tumor samples from ten different tumors. The central reference value is marked in *red*, the *blue* labels represent the seven different measurements at the seven institutes of pathology

**Fig. 3:**
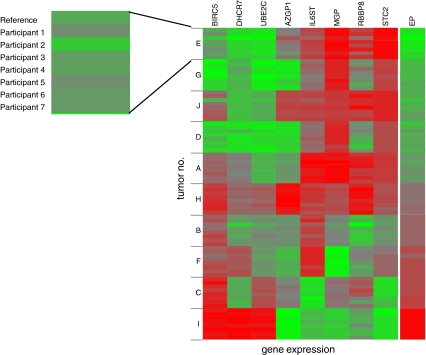
Cluster analysis of the results of the RT-qPCR in the different centers. The EndoPredict score is shown on the *right*

Therefore, the EP scores of 69 of the 70 measurements were correct with respect to the reference value and one was just at the pre-specified cutoff. For the EP score, the results of the individual measurements are shown in Fig. 2. As shown in Fig. 3, there was a high concordance in the analysis of the different genes in the seven laboratories. The EndoPredict score is arranged as a linear combination of eight genes for increased robustness. Three genes (*BIRC5*, *DHCR7*, *UBE2C*) are positively associated with the EP score, the other genes (*AZGP1*, *IL6ST*, *MGP*, *RBBP8*, *STC2*) yield to a smaller score the higher they are expressed. For details on the biological interpretation of the genes, see supplementary appendix in Filipits et al. [14].

### Variation, correlation and concordance of results—secondary study aims

**Fig. 4:**
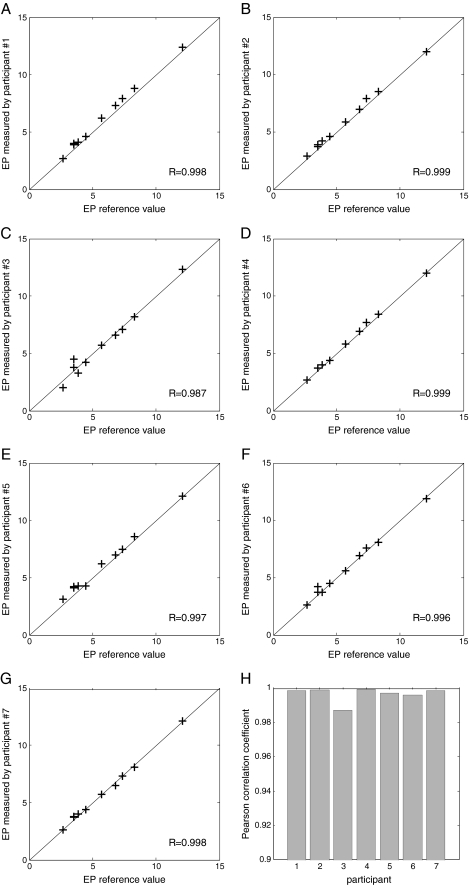
Correlation analysis of the EndoPredict test results in the seven different pathology laboratories. **a–g** Results of the individual laboratories. **h** Pearson correlation coefficients

The results of the analysis of the secondary study aims are shown in Table 2. Extraction of a sufficient amount of RNA and generation of a valid EP score was possible for all 70 study samples (100%); it was not required to ship any extra tissue sections for additional measurements. For four of the 70 samples (two EndoPredict plates), the RT-qPCR analysis had to be repeated. This was due to an instrument problem and one positive control that was out of the prespecified range. Averaging the corresponding variances per tumor block, the mean standard deviation of all 80 measurements (70 decentral measurements and ten central reference values) in ten tumor blocks was 0.25 score units. Eighty-six percent of the samples had a difference between local analysis and central reference value of 0.5 score units or less. The EP scores measured by the individual participants showed an excellent correlation with the reference values, respectively, as reflected by Pearson correlation coefficients ranging from 0.987 to 0.999 (Fig. 4). The Pearson correlation coefficient of all values compared to the reference value was 0.994. All samples were assigned to the correct EP risk group, resulting in a sensitivity and specificity of 100%, a concordance of 100%, and a kappa of 1.0.

## Discussion

In this study, we have tested the EndoPredict assay in seven different molecular pathology laboratories in Germany, Austria, and Switzerland. All laboratories fulfilled the pre-specified quality criteria and are thus qualified to run the test. For 69 of 70 samples, the quantitative EP score was measured correctly with an EP score deviation of less than 1.0 score units. Only one sample had a deviation of exactly 1.0 score unit and missed the pre-specified threshold marginally. Nevertheless, all 70 samples were assigned to the correct EP risk group (low risk or high risk).

The data demonstrate that the EndoPredict assay is a reproducible and easy-to-perform prognostic multigene expression test, which can easily be included in the routine workflow of patient care in breast cancer centers. The decentral use of the EndoPredict assay, a unique feature which has not been shown for other RNA-based multigene expression tests so far, has several advantages in comparison to centralized diagnostic services. The analysis can be integrated into the regular diagnostic workflow and the clinic–diagnostic setup in the setting of established local tumor boards with a clear interdisciplinary communication strategy. Moreover, the local pathologist can select optimal FFPE tumor material for this complex multigene assay ensuring high-quality on-site testing. Since no shipping of tumor material is necessary and the EndoPredict assay can be performed within one working day, results are promptly available for clinical decision making. Therefore, the EndoPredict assay is different from the Recurrence Score [20], which is based on an assessment in a central laboratory as well as the UPA/PAI assay [21], which requires fresh-frozen tissue.

It should be noted, however, that despite the excellent results of this proficiency testing, the standardized evaluation of quantitative RNA markers is not an easy and straightforward task. The essential elements are highly standardized reagents and controls including intensive lot-to-lot quality controls of 96-well plates coated with primers and probes as well as precisely pre-defined and technically validated limits of positive controls for quantitative RT-qPCR. Moreover, quality control was performed for individual genes-of-interest using a “noise model,” previously constructed from an independent large data set of replicate measurements [14]. The “noise model” estimates the variance of replicate-to-replicate noise from the Ct value and identifies and removes outliers within replicates. Outlier detection and removal in a complex test based on normalized expressions of several genes is crucial since outliers occur frequently and is possible only if at least three replicates for each gene are measured. Using two replicates only raises the portion of invalid test outcomes significantly, leading to a high number of repetitions of the whole EP test. Using one replicate only does not allow to detect outliers resulting in incorrect EP test results. A further reason for the robust decentral performance of the EndoPredict assay was the use of a standardized qPCR system as well as a reproducible technique to isolate RNA from FFPE tissue, which was extensively evaluated for RNA biomarker testing in previous studies [16, 18]. Finally, each participant performed a pre-defined analytical validation of the performance characteristics of the EndoPredict assay before proficiency testing.

The current evaluation needs to be continued to include other centers and additional samples to control for variation in molecular pathology laboratory standard operating procedures in different institutions and regions, and a validation programme for this is currently in preparation. While in our study, all 70 samples were assigned to the correct EP risk group; it should be mentioned that risk group assignment might vary for those samples with an EP score within 0.5 score units (2 SD) near the cutoff. In this situation, the estimated risk of distant metastasis, which is reported as a continuous parameter, might be useful for the interpretation of results. This study shows that RT-qPCR-based quantitative multigene expression analysis of FFPE tissue samples including algorithmic analysis is feasible in a decentral multicenter setting in diagnostic pathology laboratories. This opens the door for a new generation of molecular diagnostic tests in breast cancer that might add relevant information to the standard immunohistochemistry approach without being confined to globally centralized reference laboratories.
